# Stable coronary syndromes: pathophysiology, diagnostic advances and therapeutic need

**DOI:** 10.1136/heartjnl-2017-311446

**Published:** 2017-10-13

**Authors:** Thomas J Ford, David Corcoran, Colin Berry

**Affiliations:** 1 British Heart Foundation Glasgow Cardiovascular Research Centre, Institute of Cardiovascular and Medical Sciences, University of Glasgow, Glasgow, UK; 2 West of Scotland Heart and Lung Centre, Golden Jubilee National Hospital, Clydebank, UK; 3 University of New South Wales, Sydney, NSW, Australia; 4 British Society of Cardiovascular Research, Glasgow, UK

**Keywords:** cardiac computer tomographic (ct) imaging, cardiac magnetic resonance (cmr) imaging, cardiac risk factors and prevention, chronic coronary disease, pharmacology

## Abstract

The diagnostic management of patients with angina pectoris typically centres on the detection of obstructive epicardial CAD, which aligns with evidence-based treatment options that include medical therapy and myocardial revascularisation. This clinical paradigm fails to account for the considerable proportion (approximately one-third) of patients with angina in whom obstructive CAD is excluded. This common scenario presents a diagnostic conundrum whereby angina occurs but there is no obstructive CAD (ischaemia and no obstructive coronary artery disease—INOCA). We review new insights into the pathophysiology of angina whereby myocardial ischaemia results from a deficient supply of oxygenated blood to the myocardium, due to various combinations of focal or diffuse epicardial disease (macrovascular), microvascular dysfunction or both. Macrovascular disease may be due to the presence of obstructive CAD secondary to atherosclerosis, or may be dynamic due to a functional disorder (eg, coronary artery spasm, myocardial bridging). Pathophysiology of coronary microvascular disease may involve anatomical abnormalities resulting in increased coronary resistance, or functional abnormalities resulting in abnormal vasomotor tone. We consider novel clinical diagnostic techniques enabling new insights into the causes of angina and appraise the need for improved therapeutic options for patients with INOCA. We conclude that the taxonomy of stable CAD could improve to better reflect the heterogeneous pathophysiology of the coronary circulation. We propose the term ‘stable coronary syndromes’ (SCS), which aligns with the well-established terminology for ‘acute coronary syndromes’. SCS subtends a clinically relevant classification that more fully encompasses the different diseases of the epicardial and microvascular coronary circulation.

## Introduction

Ischaemic heart disease (IHD) persists as the leading global cause of death and lost life years in adults.^1^ Reductions in morbidity and mortality are not consistent across subgroups, with mortality being persistently high in younger women.^2^ Overall, stable ischaemic heart disease (SIHD) remains a worldwide public health problem of unmet need.

Stable coronary artery disease (CAD), or SIHD, refers to the syndrome of recurrent, transient episodes of chest pain reflecting demand-supply mismatch, that is, angina pectoris. In this article, we reappraise the causes of angina based on new insights into coronary pathophysiology. We focus on disorders of coronary artery function and their clinical relevance.

### Taxonomy

**Figure 1:**
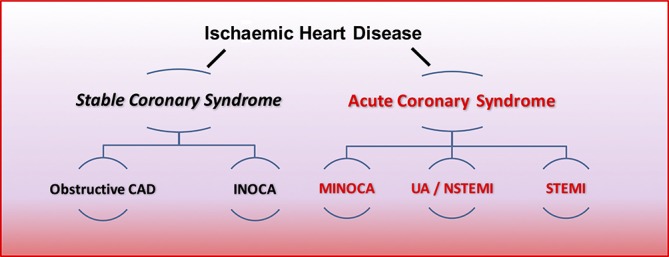
Hierarchical nomenclature of coronary artery disease endotypes that cause ischaemic heart disease. Modified with permission.^2^ CAD, coronary artery disease; INOCA, ischaemia and no obstructive coronary artery disease; MINOCA, myocardial infarction with no obstructive coronary artery disease.

Given the unmet need of IHD, recent advances in diagnostics and the need for further improvements in primary and secondary prevention, we propose the term ‘stable coronary syndromes’ (SCS) to succinctly reflect the heterogeneous pathophysiology of epicardial, microvascular and endothelial abnormalities in patients with stable angina. SCS aligns with terminology for acute coronary syndromes, and helps to standardise the hierarchy of IHD endotypes, including ischaemia with no obstructive coronary artery disease (INOCA)^3^ and myocardial infarction with no obstructive CAD (figure 1).

### The clinical conundrum of angina

Classically, angina is considered to be due to flow-limiting CAD,^4^ which by definition results in a supply-demand mismatch in myocardial perfusion. Anatomical thresholds for CAD severity vary. A widely used cut-off for obstructive CAD is taken as a stenosis of 70% in a main coronary artery (>2.5 mm) in one angiographic projection, or 50% in two projections, and 50% of the left main coronary artery.^5^ The management of patients with angina appropriately centres on the detection of obstructive epicardial CAD, which may be challenging to diagnose objectively (e.g.mild tandem lesions in series may cause flow-limiting disease). Systemic problems including anaemia and aortic stenosis also influence the propensity to angina and should be considered. In patients with obstructive epicardial CAD, the treatment involves optimal medical therapy and consideration of myocardial revascularisation with either percutaneous coronary intervention (PCI) or coronary artery bypass grafting (CABG). However, this paradigm fails to account for one-third or more patients with angina in whom obstructive CAD is excluded. A US registry of 398 978 patients referred for coronary angiography demonstrated that 39.2% of patients had no evidence of epicardial CAD.^6^ Also, angina may persist following PCI and CABG. The reasons for ‘negative’ coronary angiography are multifactorial. However, a growing body of evidence supports the use of coronary function tests, especially since a disorder of coronary artery function may be the unifying diagnosis in a patient with symptoms not explained by anatomical imaging.^7^

Historically described as cardiac syndrome X, the term coronary microvascular dysfunction (CMD) is used to describe abnormalities that result in microvascular angina (MVA). CMD is classified into five groups (table 1).^8^ The pathophysiology of CMD involves functional and/or structural abnormalities in the coronary microcirculation. MVA is prognostically important, and given the challenges in diagnosing and treating this problem in daily clinical practice, it is a condition of unmet need.^9^

**Table 1:** Classification of coronary microvascular dysfunction

Coronary microvascular dysfunction (CMD)	
Type 1	Primary CMD in the absence of underlying myocardial disease or obstructive epicardial CAD
Type 2	CMD in the presence of myocardial disease (eg, hypertrophic cardiomyopathy, hypertensive heart disease)
Type 3	CMD in the presence of obstructive CAD (either stable CAD or acute coronary syndrome)
Type 4	Iatrogenic CMD secondary to myocardial revascularisation
Type 5	CMD following cardiac transplantation

CAD, coronary artery disease.

### Pathophysiology of the coronary circulation

**Figure 2:**
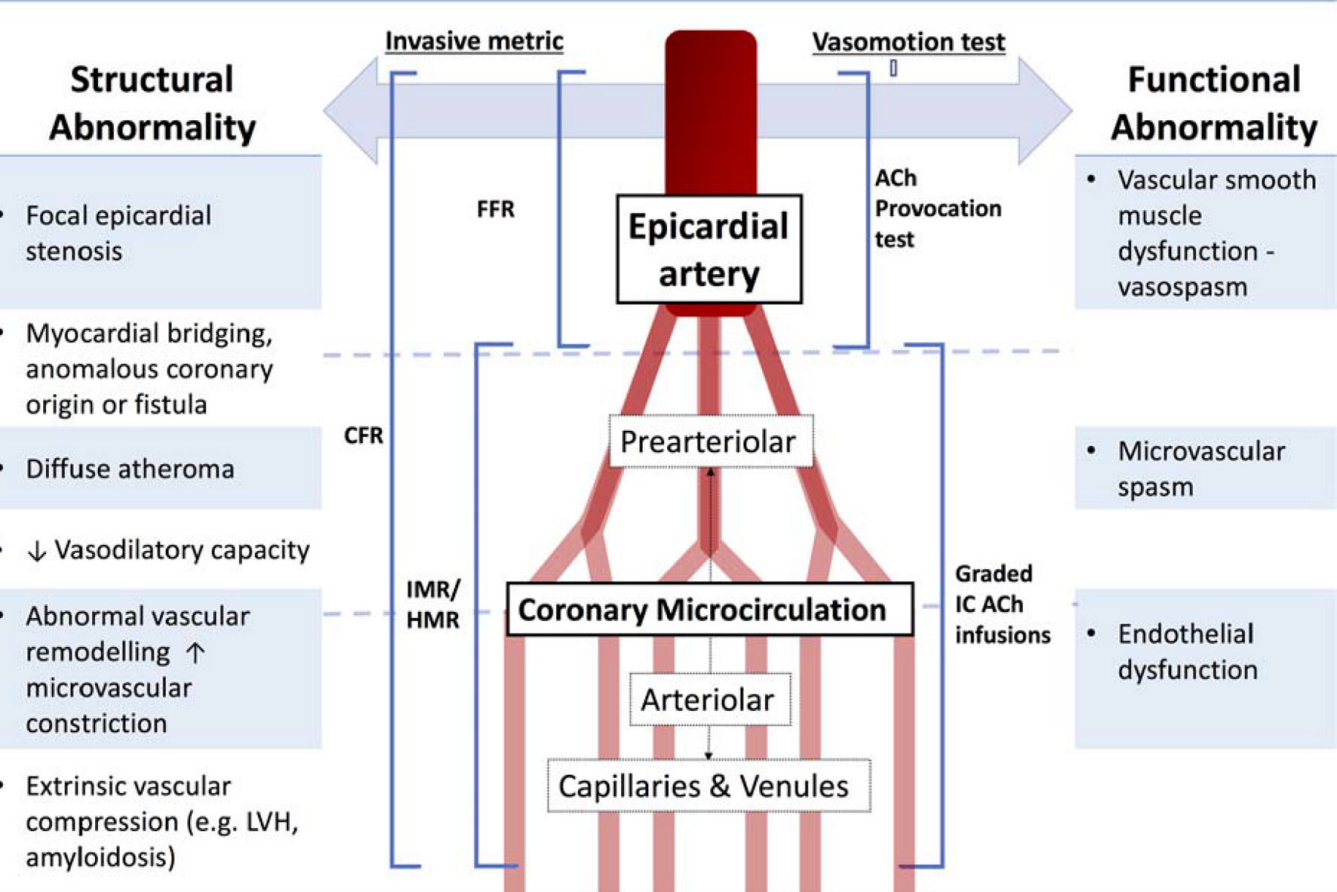
Structural and functional disorders of the coronary circulation. CFR, coronary flow reserve; FFR, fractional flow reserve; IMR, index of microcirculatory resistance; HMR, hyperaemic microvascular resistance; ACh, Acetycholine; LVH, left ventricular hypertrophy

Epicardial arteries (diameter >500 µm) are predominantly capacitance vessels and offer little resistance to flow in the healthy state. The coronary microvasculature governs resistance to myocardial perfusion. Coronary prearterioles and arterioles (vessels <500 µm) contribute approximately 25% and 50% of coronary resistance, respectively, in response to flow, stretch and metabolic stimuli.^10^ Myocardial ischaemia may result from pathophysiological processes affecting the epicardial conduit artery, the microvasculature or both (figure 2).

#### Aetiology

Cardiovascular risk factors, notably hypertension, are prevalent in patients with INOCA. Hypertension is a cause and consequence of endothelial dysfunction, atherosclerosis, microvascular remodelling, rarefaction and interstitial fibrosis. Obesity and cigarette smoking may also be relevant. Importantly, many patients with INOCA do not have risk factors for vascular disease. In these patients, the aetiology may involve a genetic abnormality, perturbations in neuroendocrine function (e.g.dysregulation of the endothelin system), autonomic nervous system abnormalities, or natural changes, such as the menopause.^3^ Finally, since the natural history of disease is rarely static, the duration and evolution (progression or remission) of disease and ageing are also relevant considerations.

#### Anatomical abnormalities in the coronary circulation

In addition to evidence-based management of obstructive atherosclerotic CAD,^4^ other structural coronary problems (including anomalous coronary vessels, coronary artery fistula, certain coronary artery bridges or aneurysms) should be considered.

Coronary microvascular disease may reflect anatomical abnormalities including microvascular remodelling (ie, reductions in capillary luminal size) and number (ie, rarefaction), and therefore increased microvascular resistance to myocardial blood flow (Poiseuille’s law). Angina may result from systemic disorders, such as hypertension, or myocardial pathology such as hypertrophic cardiomyopathy, which involves remodelling of intramural coronary arterioles, vascular rarefaction and perivascular fibrosis.^11^

In vivo, the diagnosis of anatomical changes in coronary small vessels is challenging. Yamamoto *et al* 12 performed endomyocardial biopsy in 11 patients with angina and no angiographic obstructive CAD, and demonstrated cardiomyocyte hypertrophy and replacement fibrosis compared with a control population. Osamichi *et al* 13 undertook endomyocardial biopsy in 24 patients with MVA and demonstrated smooth muscle cell hypertrophy and narrowed microvasculature due to basement membrane thickening. In contrast, Richardson *et al* 14 performed endomyocardial biopsy in seven patients with invasively diagnosed MVA and found no significant morphological abnormality.

#### Functional microvascular abnormalities

Functional abnormalities of the epicardial arteries and microvessels relate to (1) enhanced vasoconstriction, (2) impaired vasodilation secondary to endothelium-independent or endothelium-dependent mechanisms, or (3) a combination of these problems. Disorders of coronary vasomotion include epicardial and/or microvascular coronary spasm, impaired coronary artery vasorelaxation and endothelial dysfunction-related reduced myocardial blood flow.^15^ Various vasoactive substances maybe implicated. For example, endothelin-1 (ET-1) concentrations are elevated in patients with primary CMD; in 1034 patients who underwent stress positron emission tomography (PET) imaging for the investigation of angina, Johnson *et al* 16 identified abnormal diffuse heterogeneous myocardial perfusion that was associated with CMD. In an animal model, this abnormal perfusion pattern was recreated using intracoronary infusions of ET-1, implying that ET-1 contributes to abnormal vasoconstriction in patients with CMD.^17^

The coronary endothelium regulates vascular tone and myocardial blood flow via nitric oxide (NO)-dependent mechanisms.^10^ Abnormal vasoconstrictive responses to acetylcholine infusion, consistent with impaired endothelial function, occur in patients with angina and non-obstructive epicardial CAD.^15^ Abnormal endothelium-independent vasodilator function may involve resistance to NO, adenosine and prostacyclin.^18^

#### Angina-myocardial ischaemia discordance and propensity to ischaemia

The ‘ischaemic threshold’ (the heart rate–blood pressure product at the onset of angina or ECG changes) differs between individuals.^19^ Innate variations in neurogenic vascular tone and endocrine changes (eg, menopause) dictate the propensity to vasospasm while environmental factors including cold temperature, exertion and mental stress are relevant.^20^

Silent ischaemia is common and prognostically important.^21^ Interestingly, the large US CLARIFY registry highlighted the importance of symptoms, showing that angina with or without concomitant ischaemia was more predictive of adverse cardiac events compared with silent ischaemia alone.^22^ Variations in pain thresholds and cardiac innervation and diabetic neuropathy are all potential mechanisms for the discordance between symptoms and ischaemia.^23^ Patients with MVA may have abnormal adrenergic function, increased painful sensitivity to innocuous cardiac stimuli (eg, radiographic contrast), and a lower pain threshold and tolerance to the algogenic effects of adenosine (thought to be the main effector of ischaemia-mediated chest pain).^24 25^

### Advances in the diagnosis of disorders of coronary artery function

**Figure 3:**
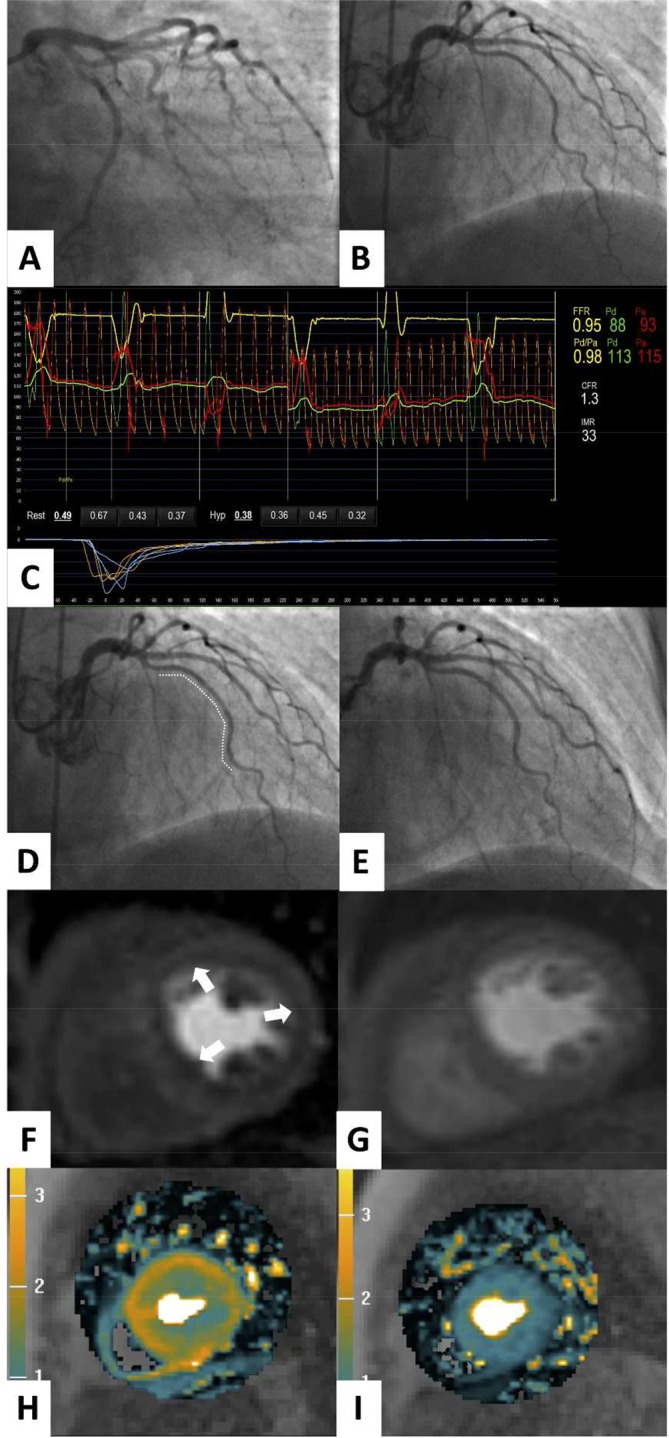
Clinical case demonstrating the utility of non-invasive and invasive diagnostic tests for coronary artery function. A 73-year-old woman presented with a 2-year history of typical Canadian cardiovascular society (CCS) class 2 angina. The patient had type 2 diabetes mellitus, an elevated body mass index and had previously been documented to have a normal invasive coronary angiogram 8 years previously. Invasive coronary angiography (A,B) demonstrated unobstructed epicardial coronary arteries. In the left anterior descending artery, the fractional flow reserve (FFR) value was 0.95, consistent with no epicardial flow-limiting stenosis (C). The coronary flow reserve (CFR) was reduced (1.3, normal >2.0), and the index of microcirculatory resistance (IMR) was elevated (33 units, normal <25), indicative of impaired epicardial and microvascular vasodilation and increased microvascular resistance respectively (C). Coronary endothelial function assessment using graded intracoronary acetylcholine infusion revealed mild vasoconstriction (dashed line) consistent with endothelial dysfunction (D) compared with endothelial-independent function testing with intracoronary glyceryl trinitrate (E). There was inducible coronary vasospasm using 100 µg acetylcholine bolus over 20 s (not shown). The patient subsequently underwent adenosine stress perfusion CMR, which demonstrated an inducible circumferential subendocardial perfusion defect in the basal short axis slice (arrows) with adenosine stress (F), compared with the corresponding rest perfusion imaging (G). A pixel-wide fully quantitative myocardial blood flow analysis confirmed markedly reduced myocardial blood flow in the subendocardium with adenosine stress (H) compared with the corresponding rest perfusion image (I). A diagnosis of coronary microvascular dysfunction was made. The patient was symptomatically improved at 3-month follow-up after treatment with nebivolol, statin and ACE inhibitors was started. The CMR methods were provided by Andrew Arai and Li-Yueh Hsu, National Institutes of Health, MD.

Coronary angiography has a resolution of 500 µm and the microvasculature is not visible on CT or invasive angiography. Myocardial biopsy is not a feasible diagnostic option. Therefore, currently, the diagnosis of CMD is empirical when specific tests of coronary function are not used (figure 3).

#### Non-invasive assessment of coronary artery function

Microvascular disease may be a generalised process resulting in diffuse myocardial perfusion abnormalities. Therefore, traditional non-invasive ischaemia tests may be normal in patients with CMD due to the absence of regional perfusion abnormalities typically seen in obstructive CAD. Myocardial perfusion scintigraphy has comparatively low spatial resolution (~1×1 cm per pixel), and is thus relatively insensitive for detection of subtle perfusion abnormalities secondary to microvascular dysfunction. Stress transthoracic Doppler echocardiography (TTDE) is typically performed in the left anterior descending, and is a potentially feasible and cheap method of assessing flow velocity at rest and during maximal hyperaemia to estimate coronary flow reserve (CFR); however, TTDE lacks accuracy and does not interrogate all myocardial segments.^26^

The reference-standard non-invasive assessment of myocardial blood flow is stress PET imaging, which permits quantitative flow derivation in mL/g/min. Clinically, PET-derived quantification of myocardial blood flowcan assist in the diagnosis of diffuse and impaired CFR, which is associated with increased risk of major adverse cardiac events (MACE).^27^ In real-world practice, the use of PET is limited by its availability (including radioisotopes), cost and exposure to ionising radiation.

Cardiac magnetic resonance (CMR) imaging holds most promise as a preferred non-invasive imaging option. Although CMR is also comparatively expensive, it has clear benefits, including lack of ionising radiation, high spatial resolution (ie, ~2.5×2.5 mm at 1.5 Tesla, ~1 ×1 mm at 3.0 Tesla), high sensitivity and specificity for perfusion abnormalities, and multiparametric imaging techniques (reference-standard left ventricular volumes and function, myocardial tissue characterisation with late gadolinium enhancement imaging and parametric mapping). Panting *et al* 28 demonstrated the qualitative detection of inducible circumferential subendocardial perfusion defects in patients with syndrome X. Semiquantitative assessment of CFR (the myocardial perfusion reserve index, MPRi) from CMR has been shown to predict abnormal response to invasive coronary reactivity testing, and is an important prognosticator in a large natural history study of women with non-obstructive CAD.^29^ Novel pixel-wise absolute perfusion quantification of myocardial perfusion by CMR will likely improve the efficiency of absolute quantification of myocardial blood flow by CMR.^30^

#### Invasive guidewire-based techniques

Invasive tests of coronary artery function are the reference standard for the diagnosis of CMD.^31^ We contend that a complete diagnostic evaluation of the coronary circulation should assess structural *and* functional pathology. Pressure-derived indices, such as fractional flow reserve (FFR), contrast-enhanced FFR, instantaneous wave-free ratio (iFR) and resting Pd/Pa, are useful tests to guide revascularisation decisions.^32^ However, as is the case with coronary angiography, these indices do not inform the clinician about microvascular resistance and or vasodilator potential.

CFR reflects the ratio of hyperaemic flow to basal flow and was first described by Gould *et al* in 1974.^33^ Microvascular resistance may be measured by thermodilution (index of microcirculatory resistance, IMR)^34^ or Doppler (hyperaemic microvascular resistance, HMR).^35^ CFR and IMR/HMR reflect distinct properties of vascular (dys)function and discordance (normal/abnormal) is common.^36^ CFR reflects the combined vasodilator capacity of the epicardial coronary artery and its subtended microvasculature. There are some limitations to using invasively measured CFR in isolation due to its sensitivity to systemic haemodynamics, myocardial contractility and challenges with establishing true resting coronary blood flow during invasive coronary angiography. Specific measures of microvascular resistance (i.e., IMR and HMR) are more reproducible, specific and are directly informative about microvascular disease.^37^

Sezer *et al* 38 assessed coronary physiology in patients with diabetes with INOCA, showing that early reduction in CFR was driven by disturbed coronary regulation and high resting flow. In long-standing diabetes, elevated microvascular resistance may reflect structural remodelling of small vessels. This process parallels the paradox of microvascular disease in diabetic nephropathy where increased glomerular filtration rate (GFR) typifies the early stages of disease prior to later structural damage and reduction in GFR.

#### Functional disorders of coronary vasomotion

**Figure 4:**
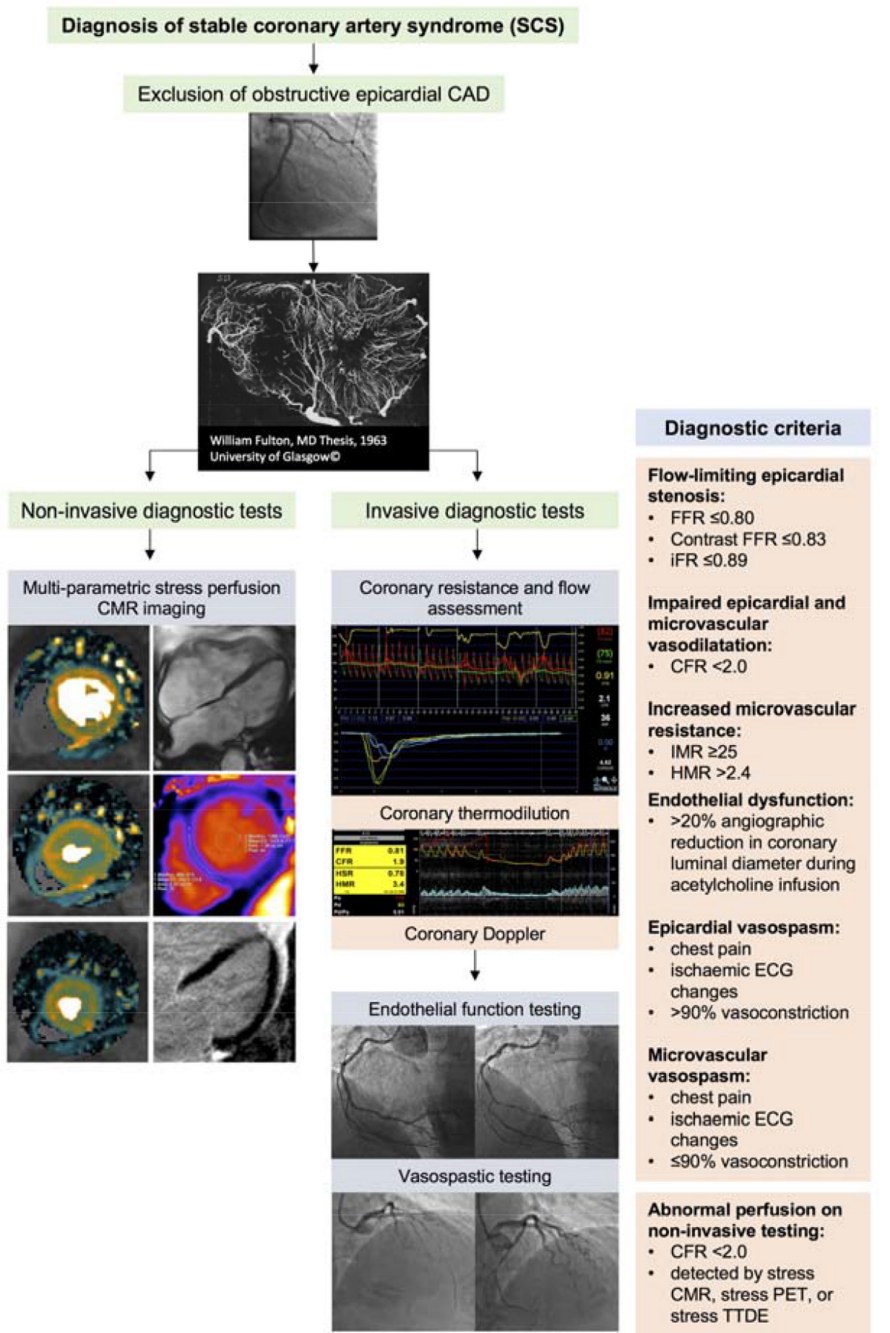
Schematic illustration of the diagnostic work-up for SCS following exclusion of obstructive epicardial CAD. (1) Non-invasive diagnostic testing with multiparametric stress perfusion CMR imaging assessment demonstrating pixel-wide fully quantitative myocardial blood flow analysis from cardiac base to apex, cine imaging, native T1 parametric mapping and late gadolinium enhancement imaging. (2) Invasive diagnostic testing with (A) dual pressure-sensitive and temperature-sensitive coronary wire or coronary Doppler and pressure-sensitive wire, and (B) endothelial and vasospastic testing with intracoronary acetylcholine. CAD, coronary artery disease; CFR, coronary flow reserve; CMR, cardiac magnetic resonance; FFR, fractional flow reserve; HMR, hyperaemic microvascular resistance; iFR, instantaneous wave-free ratio; IMR, index of microcirculatory resistance; PET, positron emission tomography; SCS, stable coronary artery syndrome; TTDE, transthoracic Doppler echocardiography.

FFR, CFR and IMR are typically derived using intravenous adenosine, which is an endothelium-independent vasodilator. Assessment of coronary endothelial function has distinct prognostic utility.^39^ Abnormal flow response to endothelial agonist can be assessed using symptoms and angiography alone (ie, >20% angiographic reduction in coronary luminal diameter during acetylcholine infusion),^31^ intracoronary Doppler flow measurement or with thermodilution. Acetylcholine may be used at higher bolus doses (eg, 100–200 µg) in a provocation test to detect abnormal coronary vasoreactivity (ie, vasospasm). A consensus document by the Coronary Vasomotion Disorders International Study Group (COVADIS) defines the criteria for a positive provocative test as meeting the following criteria: (1) reproduction of the usual chest pain, (2) ischaemic ECG changes and (3) >90% vasoconstriction on angiography^40^ (figure 4).

## Recent clinical evidence

### Detection and incidence

Lee *et al* prospectively enrolled 139 consecutive patients in a single-centre study with angina and no obstructive CAD. During comprehensive invasive multimodality assessment at angiography, all patients had atherosclerosis on intravascular ultrasound, 21% had abnormal IMR, 44% had endothelial dysfunction and only 23% had no explanation for their symptoms.^41^ Coronary vasoreactivity testing with acetylcholine is generally safe and useful for the detection of epicardial and/or microvascular spasm.^15^ The prevalence of microvascular spasm and vasospastic angina (VSA) is not fully resolved, but these conditions may occur in up to two-thirds of patients with a ‘negative’ angiogram.^42^

Coronary atherosclerosis and abnormal vasomotion are inextricably linked. A Korean study of CFR and IMR in angiographically moderate epicardial lesions demonstrated around a quarter of 516 coronary arteries had an elevated IMR and a similar proportion had reduced CFR (<2.0).^36^ Both low CFR with elevated IMR were associated with poor prognosis.

### Prognosis of patients and no obstructive CAD

The prognosis of SCS is linked with the underlying pathophysiological mechanism and varies depending on the population studied.^9^ Patients with angiographically normal coronaries and only exercise-induced symptoms may be in a better prognostic group.^43^ Data from the Women’s Ischemia Syndrome Evaluation (WISE) study suggests that there is a worse prognosis; the 5-year annualised risk of MACE was 16.0% in women with non-obstructive CAD, 7.9% in women with normal coronary arteries and 2.4% in an asymptomatic control group (p≤0.002 after adjustment for baseline cardiovascular risk).^9^ Similarly, a Danish cohort study of 11 223 patients with angina found an increased risk of MACE for patients with diffuse non-obstructive CAD and those with normal coronaries (adjusted HR of 1.85 and 1.52, respectively), compared with a reference population.

### Therapy

#### Pharmacological symptomatic therapy

A detailed review of therapy for the different disorders of coronary artery function is beyond the scope of this review.^44^ A summary of currently available therapies aligning with the different SCS disease endotypes is shown in table 2 (see additional references in online supplementary file 1). Robust evidence for the treatment of SCS is lacking. The treatment effect in many studies is potentially diluted by enrolment of heterogeneous groups of patients with distinct pathophysiological mechanisms of CMD that may respond differently to specific treatment modalities. Current European Society of Cardiology (ESC) guidelines provide recommendations for patients with CMD suggesting ß-blockers as first-line therapy, with calcium antagonists recommended if the former is not tolerated or efficacious.^4^ Unlike in patients with angina and obstructive CAD, nitrates are not generally effective for treating SCS due to CMD.^45^ In a randomised, placebo-controlled clinical trial of ranolazine led by the WISE investigators, although there were no overall improvements in angina and MPRi with ranolazine, benefit was observed in the subgroup of patients with a reduced CFR (<2.5) at baseline.^46^ Patients with VSA may benefit symptomatically from treatment with both nitrate and calcium channel antagonists while the latter may have prognostic benefit.^47^ Rho-kinase inhibitors and endothelin-receptor antagonists represent potential future therapeutic options.

10.1136/heartjnl-2017-311446.supp1 Supplementary file 1

**Table 2:** Treatment of SCS endotypes

SCS endotype	Investigation	Pathophysiology	Treatment	Efficacy	Side effects
Microvascular angina secondary to impaired vasodilation	Reduced CFR and/or increased microvascular resistance	Anatomical remodelling, vascular rarefaction, disturbed coronary regulation	ß-blockers	Reduction in myocardial oxygen consumption	Fatigue, blurred vision, cold hands
			ACE inhibitors	Improve CFR, reduce workload, may improve microvascular remodelling	Cough, renal impairment, hyperkalaemia
			Ranolazine	Improves MPRi in patients with MVA and reduced CFR	Nausea, dizziness, headache
			Phosphodiesterase inhibitors	↓cGMP degradation, ↑vascular smooth muscle relaxation and ↑ CFR for those with baseline CFR <2.5	Flushing, tinnitus, headache
Microvascular angina secondary to abnormal vasoconstriction	Hyper-reactivity to stimuli (eg, acetylcholine, exercise, stress)	Endothelial dysfunction, inappropriate prearteriolar vasoconstriction	ACE inhibitors	Improves endothelial vasomotor dysfunction	Cough, renal impairment, hyperkalaemia
			Calcium antagonists	Vascular smooth muscle relaxation, reduction in myocardial oxygen consumption	Constipation, ankle swelling, flushing
			Nicorandil	Potassium channel activator with coronary microvascular dilatory effect	Dizziness, flushing, weakness, nausea
			Statins	Improved coronary endothelial function, pleiotropic effects including reduced vascular inflammation	Myalgia, headache, cramps
			Exercise	Beneficial effect on endothelium, ↓ resting blood flow and ↑ vasodilatory capacity	Muscle fatigue, myalgia
			Hormone replacement therapy	Oestrogen therapy improves endothelial function short term in CMD	↑ Risk of breast cancer, marginally ↑ risk of CVD
Microvascular angina secondary to abnormal pain processing	Enhanced nociception	Dysfunctional cortical pain processing	Tricyclic antidepressants	Improved symptom burden potentially through reduced visceral pain	Blurred vision, dry mouth, drowsiness, impaired coordination
			Xanthine derivatives	Antialgogenic effect (due to the direct involvement of adenosine in cardiac pain generation)	Nausea and vomiting, palpitations
Epicardial and/or microvascular coronary vasospasm	Propensity to coronary vasospasm	Vascular smooth muscle hyper-reactivity	Calcium channel antagonists	↓ Spontaneous and inducible coronary spasm via vascular smooth muscle relaxation and ↓ oxygen demand	Constipation, ankle swelling, flushing
			Nitrates	↓ Spontaneous and inducible coronary spasm via large epicardial vasodilation, ↓ oxygen demand; lack of efficacy in microvascular angina with potentially deleterious effect	Headaches, dizziness, flushing
			Rho-kinase inhibitors	↓ Calcium sensitivity of smooth muscle by ↑ phosphatase activity reducing phosphorylated (active) myosin light chains	Rash, dizziness; not licensed for use in Europe or USA
Adjunctive non-pharmacological interventions	May be useful in all endotypes	Metabolic syndrome, endothelial dysfunction, cardiovascular risk factors, anxiety/depression	Smoking cessation, exercise, cardiac rehabilitation, Mediterranean diet, cognitive behavioural therapy		

CFR, coronary flow reserve; CMD, coronary microvascular dysfunction; MPRi, myocardial perfusion reserve index; MVA, microvascular angina; SCS, stable coronary syndrome; CVD, cardiovascular disease; cGMP, *Cyclic* guanosine monophosphate

#### Secondary prevention

ESC guidelines support the use of secondary prevention with aspirin and statin therapy.^4^ In contrast, the UK National Institute for Health and Care Excellence guideline-95 on management of stable angina cites the enigmatic term ‘Syndrome X’, but guidance on the diagnosis and management is limited, reflecting the limited evidence base.^48^ Contemporary guidelines should be followed for the management of vascular risk factors in patients with INOCA.^3 41^ In patients with CMD, statin therapy reduces ischaemia as revealed by reduced ST-segment deviation following exercise testing and improves exercise capacity and flow-mediated dilatation in the brachial artery in patients with CMD.^49^ ACE inhibitors (ACEi) improve endothelial dysfunction and vasoreactivity via NO stimulation helping to reverse vascular hypertrophy and improve vascular compliance.

#### Non-pharmacological therapy

Lifestyle measures promote well-being in patients with INOCA through smoking cessation, healthy eating (eg, Mediterranean diet) and physical activity.^3^ The Comprehensive Treatment of Angina in Women With Microvascular Dysfunction trial (NCT02910154) will randomise women with angina and CMD to comprehensive multimodality intervention (dietary, exercise, and statin and ACEi therapy) or control therapy to determine whether angina and microvascular dysfunction can be improved.

### Future directions

Overall, there is a critical missing link between the use of relevant diagnostic tests of coronary artery function, therapeutic agents with proven efficacy and health outcomes of patients with angina without obstructive CAD. This gap in evidence is currently being addressed in the British Heart Foundation CORonary MICrovascular Angina randomised controlled trial (CorMicA NCT03193294). A personalised approach to therapy is desirable and it is reasonable to target those patients with impairment of microvascular dilation characterised by reduced CFR with anti-anginals that reduce heart rate and oxygen consumption (eg, ß-blockers), whereas vasodilators or ACEi would be more appropriate for patients with evidence of in microvascular constriction.^50^

‘Stable coronary syndrome’ reflects a disease-based, clinically relevant classification that reflects the interaction between structural and functional disorders throughout the coronary circulation. The paucity of pathway-specific therapy presents an opportunity for novel research using stratified medicine. Our vision is for a personalised medicine approach whereby SCS endotypes, defined by the results of coronary function tests, may benefit from targeted therapy. Further research is needed to determine whether this paradigm may lead to patient benefits (table 3).

**Table 3:** Proposed comprehensive research strands for patients with ischaemia and no obstructive coronary artery disease (INOCA)

Comprehensive INOCA research strands	
Stratified medicine trials	Diagnostic tests (rule-in/rule-out) Stratification of endotypes for evidence-based therapy
Vascular science	Investigations of disease mechanisms, for example, endothelial dysfunction and dysregulation of the endothelin system
Imaging and modelling	Clinical trials of quantitative perfusion CMR versus standard methods Patient-specific computed models of disease to predict responses to novel therapeutics
Molecular pathology and vascular histopathology	Protein assay-based scores and genetic variants are potential biomarkers for disorders of coronary function. Identification of drug targets
Therapeutic trials	Enhanced system antagonists Vasodilating ß-blockers Lifestyle interventions, for example, exercise, weight loss
Health informatics and value assessments	Assess the cost-effectiveness of innovative stratified approaches
Patient and public involvement	Ensures relevance of research to patients and carers
