# Supplementary material for: Stable coronary syndromes: pathophysiology, diagnostic advances and therapeutic need
Source: Heart. 2017 Oct 13;104(4):284–92. doi: 10.1136/heartjnl-2017-311446 (PMC5861393; doi:10.1136/heartjnl-2017-311446)
Supplement: Supplementary file 1 [file heartjnl-2017-311446supp001.pdf]

SCS endotype	Investigation	Pathophysiology	Treatment	Efficacy	Side-effects	Relevant clinical trials
**Microvascular angina secondary to impaired vasodilatation**	Reduced CFR and/or increased microvascular resistance	Anatomical remodelling, vascular rarefaction, disturbed coronary regulation	**ß-blockers**	Reduction in myocardial oxygen consumption	Fatigue, blurred vision. cold hands.	Bugiardini et al[1]<br>Kayaalti et al[2]
			**ACE inhibitors**	Improve CFR, reduce workload, may improve small vessel remodelling	Cough, renal impairment, hyperkalaemia.	Pauly et al[3]
			**Ranolazine**	Improves MPRi in patients with MVA and reduced CFR	Nausea, dizziness, headache	NCT01342029[4]<br>NCT01815957[5]
			**Phosphodiesterase inhibitors**	↓cGMP degradation, ↑vascular smooth muscle relaxation and ↑ CFR for those with baseline CFR <2.5	Flushing, tinnitus, headache	Denardo et al[6]
**Microvascular angina secondary to abnormal vasoconstriction**	Hyper-reactivity to stimuli (e.g. acetylcholine, exercise, stress)	Endothelial dysfunction, inappropriate prearteriolar vasoconstriction	**ACE inhibitors**	Improves endothelial vasomotor dysfunction	Cough, renal impairment, hyperkalaemia.	Mancini et al[7]
			**Calcium antagonists**	Vascular smooth muscle relaxation, reduction in myocardial oxygen consumption	Constipation, ankle swelling, flushing	Bairey Merz et al [8]
			**Nicorandil**	Potassium channel activator with coronary microvascular dilatory effect	Dizziness, flushing, weakness, nausea	Chen et al[9]
			**Statins**	Improved coronary endothelial function, pleiotropic effects including reduced vascular inflammation	Myalgia, headache, cramps	Treasure et al[10]<br>Kayikcioglu et al[11]
			**Exercise**	Beneficial effect on endothelium ↓ resting blood flow and ↑ vasodilatory capacity	Muscle fatigue, myalgia	Czernin et al[12]
			**Hormone-replacement therapy**	Oestrogen therapy improves endothelial function short-term in CMD	↑ Risk of breast cancer, marginally ↑ risk of CVD	NCT00600106[13]
**Microvascular angina secondary to abnormal pain processing**	Enhanced nociception	Dysfunctional cortical pain processing	**Tricyclic antidepressants**	Improved symptom burden potentially through reduced visceral pain	Blurred vision, dry mouth, drowsiness, impaired coordination	Canon et al[14]
			**Xanthine derivatives**	Anti-algogenic effect (due to the direct involvement of adenosine in cardiac pain generation)	Nausea & vomiting, palpitations	Eriksson et al[15]
**Epicardial and/or microvascular coronary vasospasm**	Propensity to coronary vasospasm	Vascular smooth muscle hyper-reactivity	**Calcium channel antagonists**	↓ spontaneous and inducible coronary spasm via vascular smooth muscle relaxation and ↓ oxygen demand	Constipation, ankle swelling, flushing	Nishigaki et al meta-analysis[16]<br>Johnson et al[17]
			**Nitrates**	↓ spontaneous and inducible coronary spasm via large epicardial vasodilation, ↓ oxygen demand. Lack of efficacy in microvascular angina with potential deleterious effect	Headaches, dizziness, flushing	Lombardi et al[18]<br>Russo et al[19]
			**Rho-kinase inhibitors**	↓ Calcium sensitivity of smooth muscle by ↑ phosphatase activity reducing phosphoralated (active) myosin light chains	Rash, dizziness. Not licensed for use in Europe or USA.	Mohri et al[20]
**Adjunctive non-pharmacological interventions**	May be useful in all endotypes	Metabolic syndrome, endothelial dysfunction, cardiovascular risk factors, anxiety/depression	Smoking cessation, Exercise, cardiac rehabilitation, Mediterranean diet, cognitive behavioural therapy			Stampfer et al[21]

Web-Only References

1. Bugiardini R, Borghi A, Biagetti L, et al. Comparison of verapamil versus propranolol therapy in syndrome X. *Am J Cardiol* 1989;63(5):286-90. doi: DOI: http://dx.doi.org/10.1016/0002-9149(89)90332-9

2. Kayaalti F, Kalay N, Basar E, et al. Effects of nebivolol therapy on endothelial functions in cardiac syndrome X. *Heart Vessels* 2010;25(2):92-6. doi: 10.1007/s00380-009-1170-1

3. Pauly DF, Johnson BD, Anderson RD, et al. In women with symptoms of cardiac ischemia, nonobstructive coronary arteries, and microvascular dysfunction, angiotensin-converting enzyme inhibition is associated with improved microvascular function: A double-blind randomized study from the National Heart, Lung and Blood Institute Women's Ischemia Syndrome Evaluation (WISE). *Am Heart J* 2011;162(4):678-84. doi: 10.1016/j.ahj.2011.07.011

4. Ahmed B, Mondragon J, Sheldon M, et al. Impact of ranolazine on coronary microvascular dysfunction (MICRO) study. *Cardiovasc Revasc Med* 2017 doi: 10.1016/j.carrev.2017.04.012

5. Bairey Merz CN, Handberg EM, Shufelt CL, et al. A randomized, placebo-controlled trial of late Na current inhibition (ranolazine) in coronary microvascular dysfunction (CMD): impact on angina and myocardial perfusion reserve. *Eur Heart J* 2016;37(19):1504-13. doi: 10.1093/eurheartj/ehv647

6. Denardo SJ, Wen X, Handberg EM, et al. Effect of phosphodiesterase type 5 inhibition on microvascular coronary dysfunction in women: a Women's Ischemia Syndrome Evaluation (WISE) ancillary study. *Clin Cardiol* 2011;34(8):483-7. doi: 10.1002/clc.20935 [published Online First: 2011/07/23]

7. Mancini GB, Henry GC, Macaya C, et al. Angiotensin-converting enzyme inhibition with quinapril improves endothelial vasomotor dysfunction in patients with coronary artery disease. The TREND (Trial on Reversing ENdothelial Dysfunction) Study. *Circulation* 1996;94(3):258-65.

8. Bairey Merz CN, Pepine CJ, Walsh MN, et al. Ischemia and No Obstructive Coronary Artery Disease (INOCA): Developing Evidence-Based Therapies and Research Agenda for the Next Decade. *Circulation* 2017;135(11):1075-92. doi: 10.1161/CIRCULATIONAHA.116.024534

9. Chen JW, Lee WL, Hsu NW, et al. Effects of short-term treatment of nicorandil on exercise-induced myocardial ischemia and abnormal cardiac autonomic activity in microvascular angina. *Am J Cardiol* 1997;80(1):32-8. [published Online First: 1997/07/01]

10. Treasure CB, Klein JL, Weintraub WS, et al. Beneficial effects of cholesterol-lowering therapy on the coronary endothelium in patients with coronary artery disease. *N Engl J Med* 1995;332(8):481-7. doi: 10.1056/NEJM199502233320801

11. Kayikcioglu M, Payzin S, Yavuzgil O, et al. Benefits of statin treatment in cardiac syndrome-X1. *European heart journal* 2003;24(22):1999-2005.

12. Czernin J, Barnard RJ, Sun KT, et al. Effect of Short-term Cardiovascular Conditioning and Low-Fat Diet on Myocardial Blood Flow and Flow Reserve. *Circulation* 1995;92(2):197-204. doi: 10.1161/01.cir.92.2.197

13. Merz CN, Olson MB, McClure C, et al. A randomized controlled trial of low-dose hormone therapy on myocardial ischemia in postmenopausal women with no obstructive coronary artery disease: results from the National Institutes of Health/National Heart, Lung, and Blood Institute-sponsored Women's Ischemia Syndrome Evaluation (WISE). *Am Heart J* 2010;159(6):987 e1-7. doi: 10.1016/j.ahj.2010.03.024

14. Cannon RO, 3rd, Quyyumi AA, Mincemoyer R, et al. Imipramine in patients with chest pain despite normal coronary angiograms. *N Engl J Med* 1994;330(20):1411-7. doi: 10.1056/NEJM199405193302003

15. Eriksson BE, Sadigh B, Svedenhag J, et al. Analgesic effects of adenosine in syndrome X are counteracted by theophylline: a double-blind placebo-controlled study. *Clin Sci (Lond)* 2000;98(1):15-20. [published Online First: 1999/12/22]

16. Nishigaki K, Inoue Y, Yamanouchi Y, et al. Prognostic effects of calcium channel blockers in patients with vasospastic angina--a meta-analysis. *Circulation journal : official journal of the Japanese Circulation Society* 2010;74(9):1943-50.

17. Johnson SM, Mauritson DR, Willerson JT, et al. A controlled trial of verapamil for Prinzmetal's variant angina. *N Engl J Med* 1981;304(15):862-6. doi: 10.1056/NEJM198104093041502

18. Lombardi M, Morales MA, Michelassi C, et al. Efficacy of isosorbide-5-mononitrate versus nifedipine in preventing spontaneous and ergonovine-induced myocardial ischaemia. A double-blind, placebo-controlled study. *Eur Heart J* 1993;14(6):845-51. [published Online First: 1993/06/01]

19. Russo G, Di Franco A, Lamendola P, et al. Lack of effect of nitrates on exercise stress test results in patients with microvascular angina. *Cardiovasc Drugs Ther* 2013;27(3):229-34. doi: 10.1007/s10557-013-6439-z

20. Mohri M, Shimokawa H, Hirakawa Y, et al. Rho-kinase inhibition with intracoronary fasudil prevents myocardial ischemia in patients with coronary microvascular spasm. *Journal of the American College of Cardiology* 2003;41(1):15-9.

21. Stampfer MJ, Hu FB, Manson JE, et al. Primary prevention of coronary heart disease in women through diet and lifestyle. *N Engl J Med* 2000;343(1):16-22. doi: 10.1056/NEJM200007063430103
